# Supplementary figures and images for: Robotic Surgery and Functional Esophageal Disorders: A Systematic Review and Meta-Analysis
Source: J Pers Med. 2023 Jan 27;13(2):231. doi: 10.3390/jpm13020231 (PMC9966072; doi:10.3390/jpm13020231)

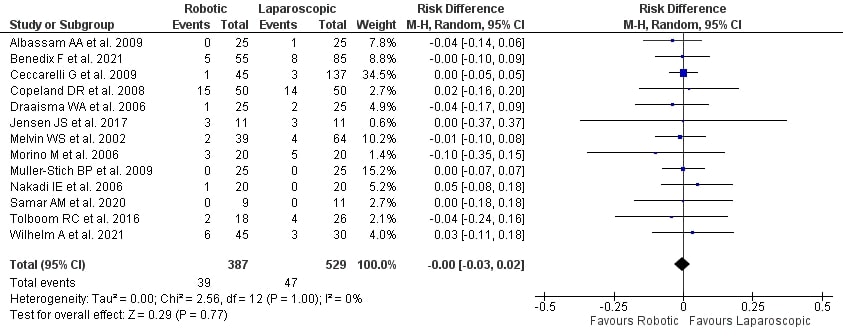

Supplement: Supplementary file 1 [file jpm-13-00231-s001.zip › Figure S1.jpg]

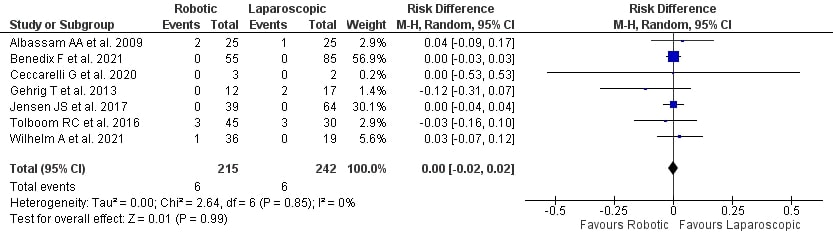

Supplement: Supplementary file 1 [file jpm-13-00231-s001.zip › Figure S2.jpg]

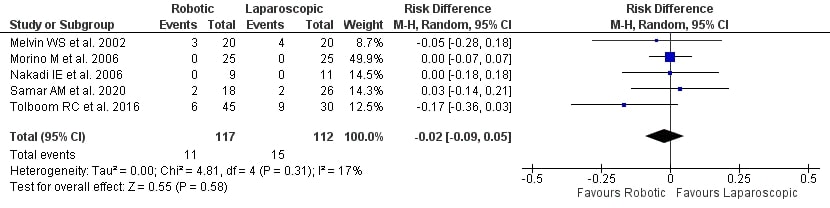

Supplement: Supplementary file 1 [file jpm-13-00231-s001.zip › Figure S3.jpg]

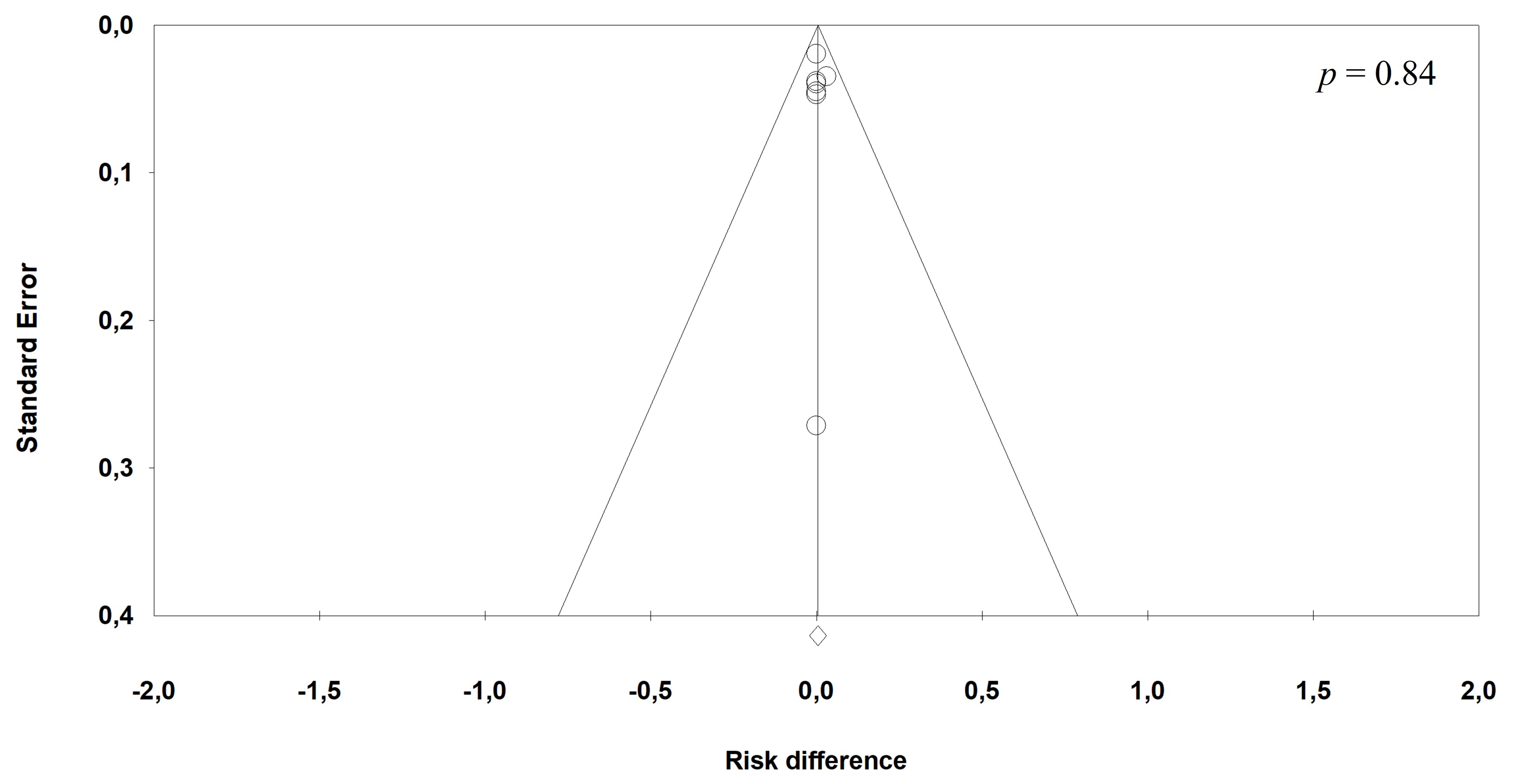

Supplement: Supplementary file 1 [file jpm-13-00231-s001.zip › Figure S4.jpg]

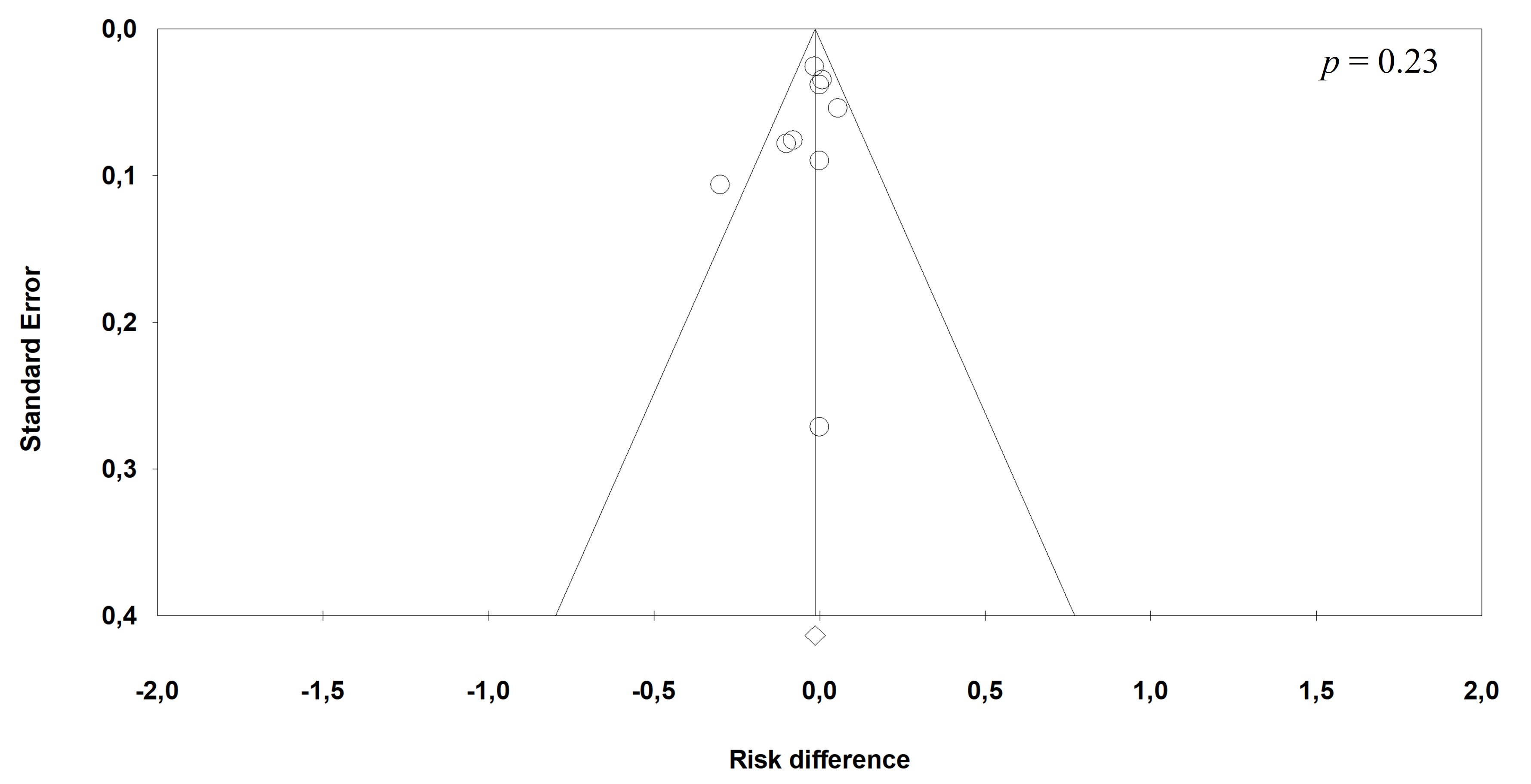

Supplement: Supplementary file 1 [file jpm-13-00231-s001.zip › Figure S5.jpg]

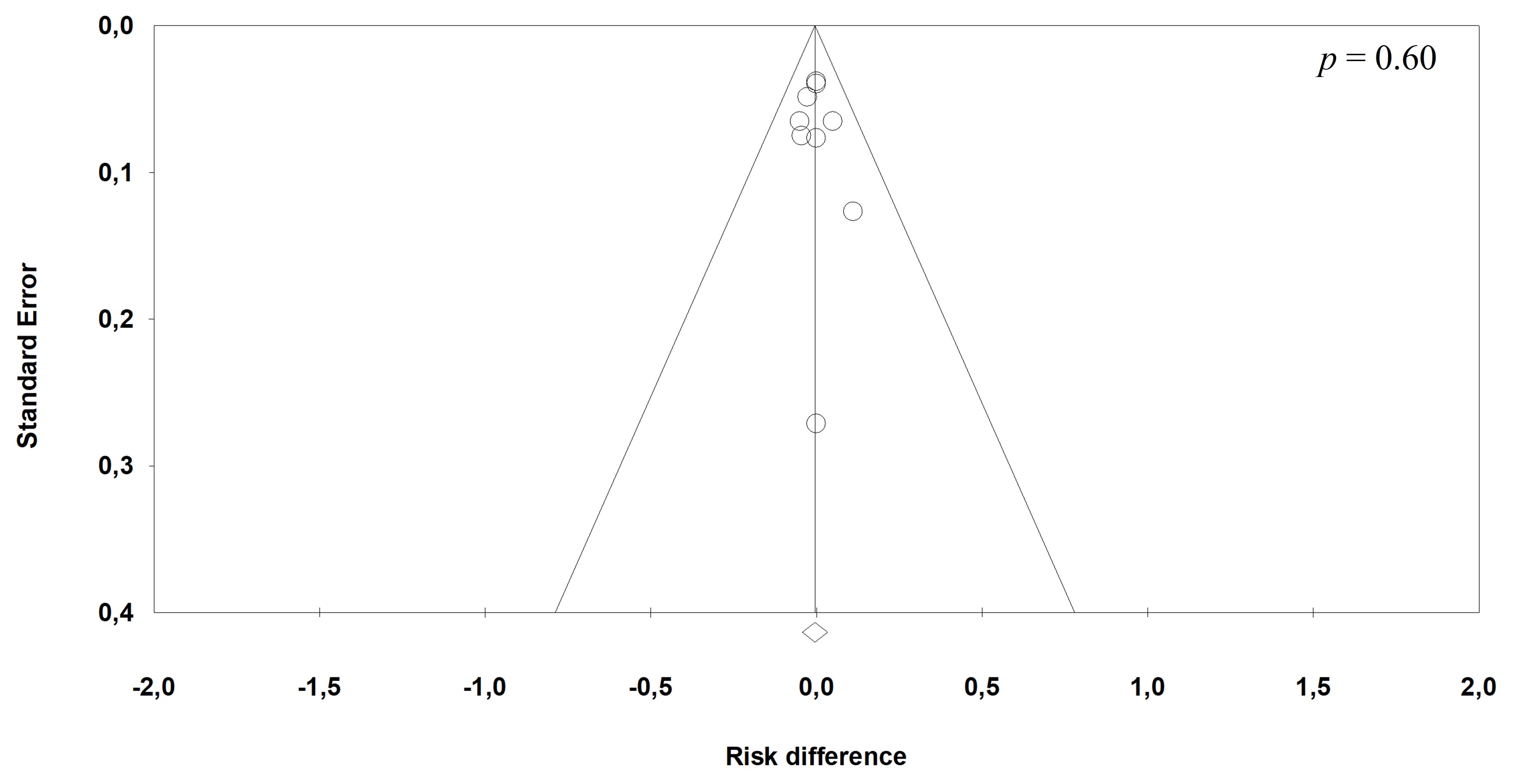

Supplement: Supplementary file 1 [file jpm-13-00231-s001.zip › Figure S6.jpg]

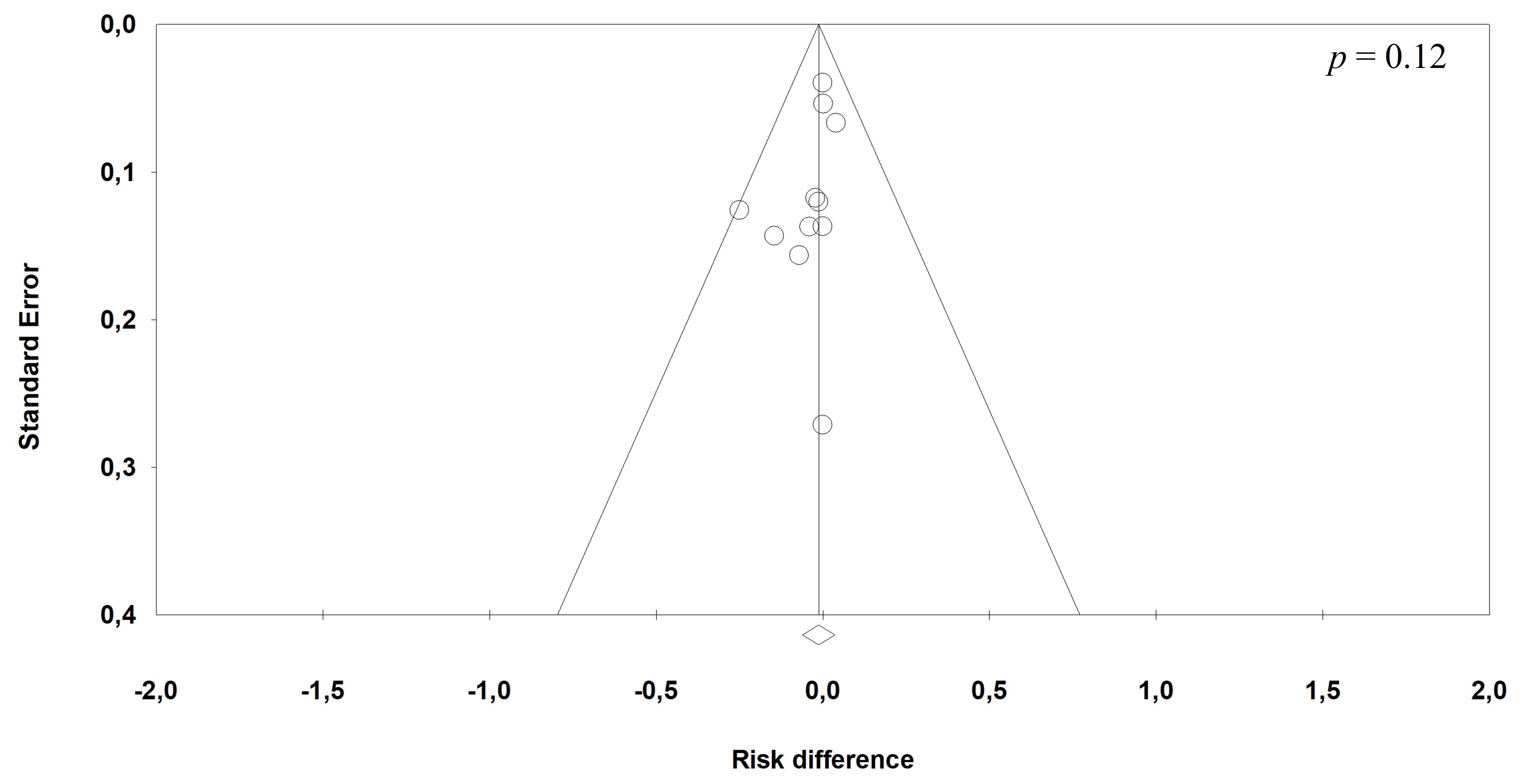

Supplement: Supplementary file 1 [file jpm-13-00231-s001.zip › Figure S7.jpg]
